# Differential roles of dopamine D1 and D2 receptors in pain modulation: a review

**DOI:** 10.3389/fneur.2026.1802976

**Published:** 2026-05-28

**Authors:** Minjie Huang, Xuejiao Li, Yingying Miao, Wenfeng Mao, Chenhao Xu, Mingxi Gu

**Affiliations:** Department of Human Anatomy, Basic Medical Sciences College, North Henan Medical University, Xinxiang, Henan Province, China

**Keywords:** anterior cingulate cortex (ACC), dopamine D1 receptor, dopamine D2 receptor, nucleus accumbens (NAc), pain modulation

## Abstract

Dopamine D1 and D2 receptors (D1R, D2R) are widely distributed in the central nervous system and play important yet distinct roles in pain regulation. Although their involvement in reward, motivation, and emotional processing is well established, a systematic understanding of their region-specific and subtype-specific functions in pain remains incomplete. This review integrates research from the past decade on four core pain-related brain regions: the prefrontal cortex (PFC), nucleus accumbens (NAc), anterior cingulate cortex (ACC), and amygdala. Existing evidence shows that D1R and D2R exhibit significant regional heterogeneity and functional complexity. In the PFC, D1R has been implicated in pain signal integration and salience encoding in some studies, while D2R shows a context-dependent regulatory role; however, the literature remains heterogeneous, and causal evidence is limited. In the NAc, D1R may mediate the intersection of endogenous and exogenous analgesic signals and may participate in pain-reward balance; D2R has been shown to modulate inflammatory pain and neuropathic pain, opioid synergy, and stress analgesia. In the ACC, D1R appears to provide tonic suppression under physiological conditions, but its function may diminish in chronic pain, potentially contributing to hyperalgesia maintenance. Conversely, activation of D2R has been reported to suppress pain symptoms and to restore inhibitory control in some studies. In the amygdala, D1R exhibits region- and cell-type specific effects, while D2R within the central amygdala may serve as an important mediator of the VTA-CeA reward-analgesia pathway. More importantly, the interaction between D1R and D2R presents patterns of functional synergy, functional antagonism, and state-dependent reorganization, which together precisely regulate the sensory and emotional dimensions of pain. Treatment targeting the dopamine receptor system is a promising pain management strategy. However, many challenges remain in achieving precise treatment targeting specific regions and cell types. Additional clinical studies are needed in the future to evaluate the efficacy of this approach in patients with chronic pain and mood disorders.

## 1 Introduction

Pain is a complex, multidimensional experience characterized by sensory discrimination and emotional motivation, and is frequently accompanied by negative emotions such as anxiety and depression that significantly impair patients' quality of life and social functioning (1, 2). Currently, opioid-based analgesics present substantial limitations, including a narrow therapeutic index, inconsistent efficacy, adverse side effects such as tolerance, dependence, and addiction, and restricted long-term applicability (3). Consequently, the development of non-opioid analgesic strategies has emerged as a significant clinical challenge. The mesolimbic dopaminergic system, a key regulator of pain processing, mediates signaling pathways that not only enhance opioid analgesia but also mitigate associated adverse effects, thereby presenting a novel avenue for pain management (4–6). Dopamine, as a principal neurotransmitter within the midbrain reward circuit, primarily exerts its effects through D1-like and D2-like receptors (7, 8). These receptors exhibit differential expression in critical pain-processing brain regions such as the anterior cingulate cortex (ACC), nucleus accumbens (NAc), and amygdala, where they dynamically modulate pain and pain-related emotions. This suggests that dopamine receptor subtypes may play distinct roles in the modulation of pain (9–12).

Existing studies have preliminarily elucidated the distinct roles of D1 and D2 dopamine receptors in pain-related brain regions (13–15). However, substantial research gaps remain. First, the cellular and molecular mechanisms underlying D1 receptor-mediated pain regulation are poorly understood, and the synergistic or antagonistic interactions between D1 and D2 receptors remain largely unexplored (16, 17). Second, the functional dichotomy of D1/D2 receptors in the medial shell of NAc has not been integrated with findings from other NAc subregions (18). Third, the specific roles and downstream signaling pathways of D1/D2 receptors in the anterior cingulate cortex (ACC)—a key hub for pain modulation—remain undefined (19). Furthermore, the synergistic mechanisms between the ACC and the prefrontal cortex (PFC), where D1/D2 receptors exhibit differential expression in chronic pain and contribute to pain tolerance and cognitive appraisal, have yet to be explored. Fourth, most studies have focused on single brain regions or receptor subtypes, lacking cross-regional integration; and the D1 receptor-mediated pain effects in the PFC and amygdala remain controversial (20, 21). Fifth, existing reviews have not systematically summarized the characteristics of D1/D2 receptors in pain modulation across the CeA, PFC, NAc, and ACC (22, 23).

**Figure 1:**
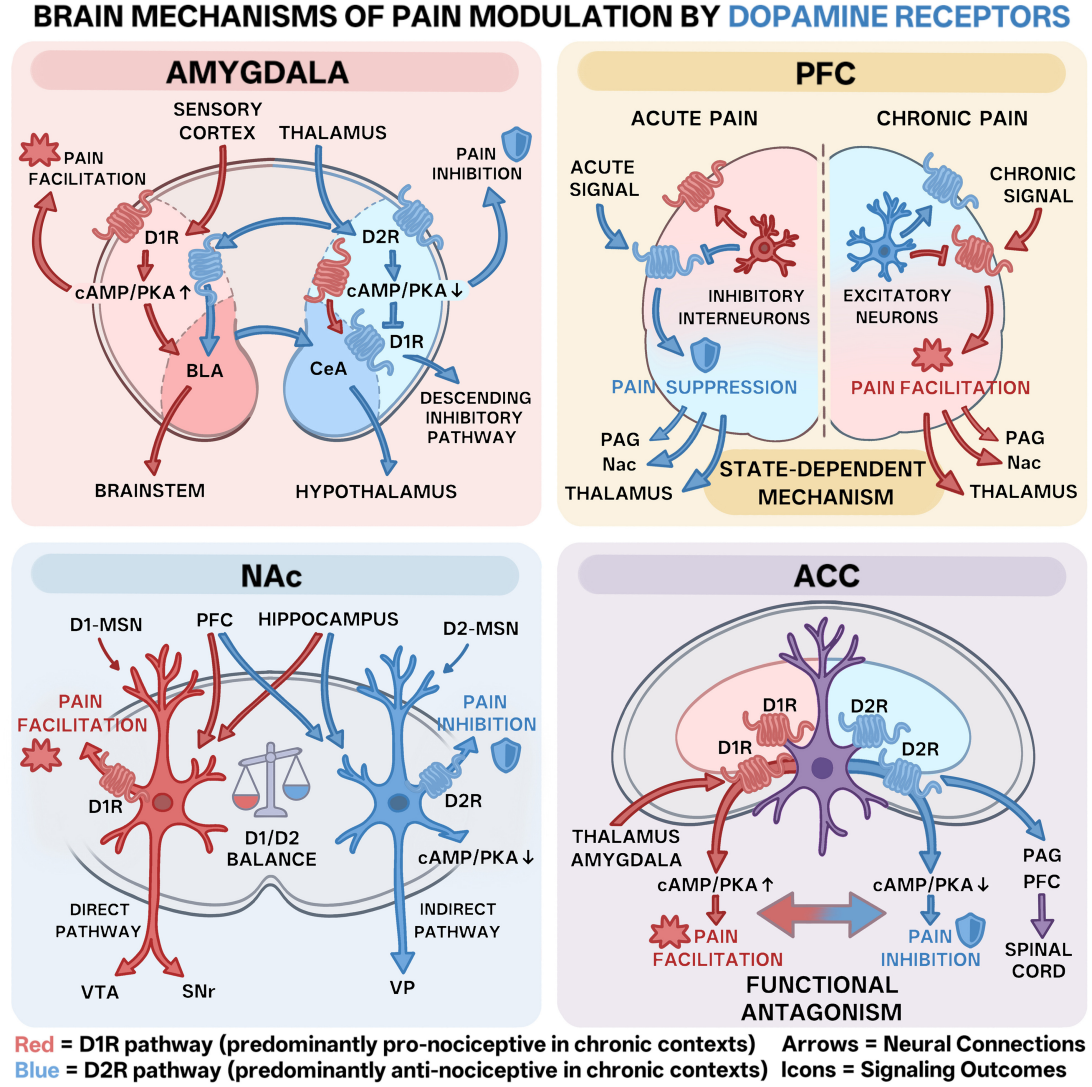
Brain mechanisms of dopamine receptor-regulated pain. This figure comprehensively illustrates the potential bidirectional regulatory roles of dopamine D1 and D2 receptors in pain perception across different brain regions. The key components include: (1) In the amygdala, D1R may enhance pain by increasing basal lateral amygdala (BLA) activity *via* the cAMP/PKA pathway, while D2R may suppress central amygdala activity to potentially produce analgesic effects; (2) The prefrontal cortex (PFC) likely modulates pain signal transmission in the periventricular gray (PAG) and nucleus accumbens (NAc) through a balance of excitatory and inhibitory neurons during acute and chronic pain states; (3) In the nucleus accumbens (NAc), D1-MSN and D2-MSN may regulate pain *via* direct and indirect pathways respectively, with their equilibrium influencing pain output in downstream brain regions (e.g., ventral tegmental area [VTA], substantia nigra reticulata (SNr), and ventral pallidum [VP]); and (4) In the anterior cingulate cortex (ACC), D1R and D2R may exert functional antagonism to promote or inhibit pain signal transmission to the thalamus, amygdala, and spinal cord, respectively. Arrows in the figure indicate neural connectivity, and icons represent signal transduction outcomes.The schematic is based on cited references; detailed experimental evidence is provided in the corresponding text sections.

This review focuses on chronic pain and its management. The central premise guiding this review is that dopamine signaling is primarily implicated in maintaining chronic pain states, rather than merely alleviating acute pain episodes. Accordingly, we narrow our scope to the four brain regions where dopaminergic dysfunction has most consistently been linked to pain chronification—the PFC, NAc, ACC, and amygdala—and examine, for each region, which specific pain dimension (sensory, affective-motivational, or cognitive) is regulated by D1R and D2R signaling. Figure 1 provides a comprehensive schematic of the potential bidirectional regulatory roles of D1R and D2R across these regions, illustrating neural circuits (arrows) and signaling outcomes (icons).

## 2 The dopamine system in pain regulation

As a key neuromodulatory system, dopaminergic pathways are essential for regulating reward processing, motivation, and affective states. Dysregulation of this system is associated with various neuropsychiatric disorders (24). In pain research, the analgesic potential of dopaminergic drugs was first systematically demonstrated by Dennis and Melzack (25). Subsequent evidence has highlighted the pivotal role of the mesolimbic dopamine pathway, which originates from ventral tegmental area (VTA) neurons and projects to key regions including the NAc, PFC, ACC, and CeA (26). Clinical neuroimaging studies have revealed characteristic neuroadaptations in multiple brain regions of chronic pain patients. Specifically, reduced VTA activity and decreased NAc gray matter volume have been observed (27, 28), whereas ACC hyperactivity and PFC thinning are also common adaptive changes (29). In addition, abnormal ACC-CeA connectivity is associated with pain persistence (30), and NAc-PFC connectivity has been proposed as a potential biomarker for pain chronicity (31, 32). Furthermore, these neuroadaptations may increase the risk of opioid dependence in chronic pain patients (33).

At the molecular level, chronic pain induces substantial dopaminergic dysregulation, characterized by three principal features: (1) diminished presynaptic dopamine synthesis, (2) reduced D2/D3 receptors availability, and (3) omdulation of pain sensitivity by polymorphisms in dopamine clearance genes (COMT, DAT) (34, 35). These changes are directly linked to core affective symptoms, such as anhedonia and motivational deficits. Preclinical studies have demonstrated that targeted modulation of NAc medium spiny neurons, PFC-NAc circuitry, or VTA activity using optogenetic or chemogenetic methods effectively alleviates both mechanical allodynia and depressive-like behaviors in neuropathic pain models (36, 37). Collectively, these findings suggest that chronic pain induces maladaptive changes in mesolimbic dopaminergic signaling (34). Dopamine deficiency may disrupt endogenous analgesic pathways and impair reward processing, thereby maintaining pain chronicity (38–40). Epigenetic modifications (e.g., DNA methylation and histone acetylation) in reward-related brain regions have been shown to contribute to pain maintenance by inducing lasting transcriptional changes (41). Importantly, the blunted reward sensitivity to opioid observed in pain states appears to be mediated by midbrain limbic dysfunction, as exemplified by upregulation of TNF-α pathway or dysregulation of RGS9-2, which modify morphine's rewarding effects (42, 43). These findings reveal potential dopaminergic-opioidergic interactions underlying pain-related addiction vulnerability.

D1 and D2 receptors are also found on peripheral sensory neurons and immune cells. Studies have shown that under certain conditions, these receptors may be involved in the transmission of pain signals and the generation of inflammatory pain (44). However, their effects in the periphery appear to be context-dependent, and their detailed mechanisms are less fully understood than those in the central nervous system (45, 46).

## 3 Amygdala

The amygdala is a central hub for processing the emotional-motivational aspects of pain, including fear, aversion, and the affective valence of nociceptive stimuli (47). As illustrated in Figure 1 (top panel), the amygdala consists of the basolateral amygdala (BLA) and the central amygdala (CeA) (48–50). D1R in the BLA may enhance pain *via* the cyclic adenosine monophosphate/protein kinase A (cAMP/PKA) pathway, whereas D2R in the CeA is proposed to suppress pain (14, 51, 52).

### 3.1 The role of D1R in the amygdala

As the “gateway” to the amygdala, the BLA is responsible for processing pain-related environmental signals and emotion-related memories. Recent studies have found that there is a group of neurons in the BLA that encode the negative affect caused by pain. These neurons are critical for generating aversion to pain (53, 54). Consistent with the schematic in Figure 1, D1R may enhance BLA activity through the cAMP/PKA pathway, thereby potentially contributing to pain sensation (51). Evidence from different behavioral domains suggests that D1R in the BLA play a role in linking environmental cues to emotional valence. For example, intra-BLA infusion of the D1R antagonist SCH23390 suppresses drug-seeking behavior in rats, and this effect persists even after a contextual shift (55), suggesting that D1R in the BLA is involved in regulating motivated behaviors relevant to both pain and reward. In contrast, the role of D1R in the CeA—main output nucleus of the amygdala—is more complex and remains debated, likely due to the high cellular heterogeneity within this region (56). Some studies support a pronociceptive role, whereas others indicate that D1R activation in the CeA may inhibit pain-responsive CeA neurons (57). Using TRAP labeling to target neurons responsive to noxious pinch, Heuermann and Gereau demonstrated that bath application of the D1R agonist SKF-38393 significantly reduced input resistance and evoked firing while increasing rheobase in these cells, and selectively suppressed NMDA-(but not AMPA-) mediated synaptic transmission from the BLA (14). These findings suggest that D1R signaling in the CeA dampens pain-related activity and may serve an **a**nalgesic function. Notably, recent studies have revealed functional heterogeneity among neuronal subpopulations in the CeA, including distinct groups expressing corticotropin-releasing factor (CRF) and somatostatin (SST) (58). As noted by Wilson et al., these cell types differ significantly in their intrinsic excitability, morphological characteristics, and input–output connections (57). Therefore, it is plausible that D1R are predominantly expressed on specific neuronal subsets, such as CRF-positive neurons. Consequently, activation of D1R may selectively enhance the negative-affective components of pain by driving activity in these specialized circuits, whereas D1R blockade could provide targeted inhibition of this pathway.

### 3.2 The role of D2R in the amygdala

As depicted in Figure 1, D2R in the CeA plays a key role in pain suppression. The CeA is mainly composed of γ-aminobutyric acid (GABA) neurons, and receives dopaminergic projections from the VTA (57). These projections can mediate the transmission and regulation of pain-related signals by activating D2R in CeA (14, 15, 59). Among these projections, VTA-CeA reward loop is an important neural pathway involved in pain relief, and D2R serves as a key receptor mediating the analgesic effect of this loop, specifically participating in pain relief regulated by VTA-CeA pathway (15). D2R can modulate pain by inhibiting the abnormal activity of pain-responsive neurons in the CeA. Figure 1 indicates that D2R (not D1R) in the CeA is primarily associated with pain suppression, while D1R in the CeA may have variable effects. Patch-clamp electrophysiological studies have shown that quinpirole, a specific D2R agonist, significantly reduces the input resistance of pain-responsive CeA neurons, decreases their evoked firing frequency, and increases the rheobase current. In addition, it inhibits NMDA receptor-mediated components of excitatory postsynaptic currents induced by the lateral amygdala stimulation (14, 52). These findings suggest that D2R effectively suppresses overactivation of pain-related CeA neurons through both synaptic and non-synaptic mechanisms, thereby exerting an analgesic effect.

Related studies have confirmed that CeA D2R participates in the formation and expression of morphine-induced conditioned place preference, suggesting that it may indirectly regulate chronic pain by integrating the rewarding effect of pain relief (59). Across different pain models, the regulatory effect of CeA D2R appears notably consistent. In a chronic inflammatory pain model, the D2R agonist pramipexole significantly reduced mechanical pain sensitivity in male rats, suggesting that D2R activation alleviates chronic inflammatory pain (52, 59). It is worth noting that the CeA exhibits dual opposing functions in pain modulation, and D2R-mediated signaling represents an important regulatory branch. At the same time, pain modulation by the CeA also displays significant hemispheric lateralization, which provides a structural basis for the region-specific regulation of D2R in different CeA subregions (60). Dysfunction of CeA D2R is also closely associated with individual differences in pain and chronic pain progression. Previous studies have shown a significant correlation between striatal D2/D3 receptors availability and individual pain responses, and abnormal dopaminergic transmission in the ventral striatum of patients with chronic back pain has been observed, indirectly reflecting the importance of dopamine receptor dysfunction in limbic system including the CeA, during chronic pain process (61).

## 4 Prefrontal cortex

The prefrontal cortex (PFC), a central hub for higher-order cognitive control, plays a critical role in pain processing—particularly in modulating its affective and cognitive dimensions (62, 63). As shown in Figure 1 (second panel), the PFC modulates pain signal transmission to the periaqueductal gray (PAG) and the NAc through a balance of excitatory and inhibitory neurons, and this balance changes between acute and chronic pain states (64, 65).

### 4.1 The role of D1R in the PFC

Accumulating evidence indicates that D1R in the PFC modulates synaptic plasticity and neuronal excitability, and participates in both acute and chronic pain states (12, 66, 67). Under acute nociceptive stimulation, the midbrain limbic dopamine system is rapidly activated, causing increased dopamine release in the PFC, which mainly modulates cortical reactivity *via* D1R (31). During the transition to chronic pain, the PFC frequently undergoes structural and functional degeneration, including neuronal atrophy, synaptic loss, and disrupted network activity, with dysregulation of D1R signaling considered a pivotal contributor (68–70). Wang et al. (71) showed that in neuropathic pain models, dopamine release in the PFC is persistently reduced, accompanied by decreased D1R–mediated synaptic excitability and reduced dendritic complexity of pyramidal neurons (19, 71, 72). This hypoactive state of D1R is closely linked to impaired cognitive flexibility and the expression of conditioned place avoidance in animals with persistent pain (73). Importantly, chronic pain-induced D1R dysfunction exhibits cell-type and circuit specificity (19, 71). Selective activation of D1R-expressing neurons in the PFC alleviates anxiety-like behaviors and anhedonia associated with neuropathic pain, suggesting that restoring PFC D1R activity may enhance inhibitory control over limbic structures such as the CeA, thereby improving emotional comorbidities of pain (74, 75). Figure 1 further illustrates that the PFC exerts top-down control over the NAc and CeA *via* glutamatergic projections; D1R signaling within the PFC modulates this control (76, 77).

### 4.2 The role of D2R in the PFC

Chronic pain leads to significant changes in the dopaminergic environment of the PFC, including abnormal dopamine release and D2 receptor signaling in the medial prefrontal cortex (mPFC) (66, 70). Studies in rodent models have shown that the dopaminergic input from the VTA to the mPFC undergoes functional changes during the development of chronic pain. Pharmacological evidence has further demonstrated that activation of dopamine receptors in the mPFC can robustly modulate mechanical nociceptive responses in naive animals, with studies implicating D2-like receptors in this effect (11, 64, 66). This finding is consistent with observation in other brain regions such as the NAc. In neuropathic pain models, D2 receptor activation has been shown to exert analgesic effects (78). However, the function of D2 receptor in the PFC does not simply follow the rule that “inhibition equals analgesia”. Previous studies have shown that dopamine regulates the excitability of PFC pyramidal neurons through D1 and D2 receptors, and dopamine receptor activation can modulate neuronal excitability *via* cAMP–PKA pathway (79). However, the functional effect of D2 receptor activation may depend on the specific cell types and neural circuits involved (80). Dopaminergic regulation in the PFC exhibits fine-tuned, context-dependent characteristics, reflecting the complexity of its receptor distribution and signal transduction mechanisms (81).

A deeper contradiction exists between cognitive function and analgesic effect. Moderate D2 receptor activation can alleviate pain-related anxiety and attentional bias by regulating hyperactive neural circuits in the PFC (80). However, excessive D2 receptor stimulation in the PFC may impair PFC-dependent cognitive functions (such as working memory and behavioral flexibility), which is consistent with research findings on cognitive deficits caused by abnormal dopaminergic regulation (81, 82). The PFC exerts “top-down” control over subcortical regions *via* its efferent projections (see Figure 1, which shows PFC outputs to PAG and NAc). In pain mechanisms, the PFC-NAc pathway is a key neural circuit involved in endogenous pain regulation; inhibition of this corticostriatal pathway aggravates sensory and emotional symptoms of acute and chronic pain (36). In addition, PFC-CeA pathway is known to be regulated by dopamine, with D2 receptor playing a modulatory role in CeA-PFC interaction (83). In chronic pain state, dysfunction of PFC D2 receptor may lead to the dysregulation of the NAc, interfere with reward processing, and result in anhedonia, consistent with the current understanding of mesocorticolimbic dopamine circuit dysfunction in chronic pain (84).

## 5 Nucleus accumbens

The nucleus accumbens (NAc) integrates pain sensory information with motivational and reward processing (23). As illustrated in Figure 1 (third panel), the NAc contains two main populations of medium spiny neurons: D1-MSNs (direct pathway) and D2-MSNs (indirect pathway), which project to downstream targets including the VTA, substantia nigra reticulata (SNr), and ventral pallidum (VP) (85–87). Their equilibrium influences pain output.

### 5.1 The role of D1R in the NAc

Studies have shown that the analgesic effect induced by VTA injection of orexin A depends on D1R activation and can be blocked by local administration of SCH-23390 (88). This regulation does not operate in isolation. In stress-induced analgesia (SIA), D1R and D2R co-regulate, and D1R antagonist can attenuate the analgesic effect produced by stress stimulation (89). In addition, a functional interaction exists between D1R and the μ-opioid receptor. Opioids can release dopamine from the VTA *via* MOR, promote NAc dopamine release, enhance downstream D1R signaling, and achieve synergistic analgesia, suggesting that D1R is positioned at the intersection of the reward-analgesia loop (90). As shown in Figure 1, D1-MSNs in the NAc project *via* the direct pathway to the VTA and SNr, and their activation has been associated with pain facilitation in some models. However, when acute pain transitions to a chronic state, the functional homeostasis of D1R faces fundamental challenges. In animal models of chronic neuropathic and inflammatory pain, the dopaminergic tone in the NAc is continuously low, leading to a significant attenuation of D1R signaling efficiency (78). Interestingly, this functional decline is not due to changes in total D1R expression, but rather to a decrease in downstream signal coupling efficiency; in a chemically induced neuropathy model, D1R-mediated adenylate cyclase activity in the NAc core region was also found to be weakened (91). These findings suggest that D1R dysfunction may play a role in the pathophysiology of chronic pain, although conclusive evidence of causality is currently lacking. It is worth noting that the functional differentiation of D1R in NAc different subregions is remarkable: D1R in the shell region is involved in coding the emotional–motivation of pain and mediates the formation of conditioned aversive memory, while D1R in the core region is responsible for the integration and transmission of pain sensory signals (18, 86). The latter is involved in the maintenance of chronic orofacial pain, the formation of pain–depression comorbidity, and the development of morphine analgesia tolerance and sensitization (18, 92, 93).

### 5.2 The role of D2R in the NAc

In the regulation of inflammatory and acute pain, D2R activation in the NAc may exert significant analgesic effects. Studies have confirmed that microinjection of the D2 agonist quinpirole inhibits formalin-induced persistent pain in a dose-dependent manner, and this effect can be blocked by the D2 antagonist raclopride or sulpiride, whereas D1 receptor agonists have no such effect, thereby clarifying the specific role of NAc D2R in mediating inflammatory pain inhibition (94). In addition, the dopamine D2 receptor exerts a dual regulatory effect on opioid analgesia. D2 receptor in the whole brain inhibits opioid analgesia, while D2 receptor in the NAc synergistically regulate acute pain and enhance the analgesic effect of morphine, suggesting their potential value as a joint intervention target for chronic pain (6). In neuropathic pain models induced by chemical agents or sciatic nerve ligation, the expression of the D2R long isoform and the activation level of G protein in the NAc core region are significantly decreased, suggesting that D2R signaling attenuation is involved in the pathological process of chronic neuropathic pain (91). D2-MSNs may regulate pain *via* direct and indirect pathways, with their equilibrium influencing pain output in downstream brain regions (see Figure 1 for the NAc → VTA/SNr/VP circuits). Activation of D2R can reverse neuropathic pain-related abnormalities. Optogenetic inhibition of NAc D2R-positive neurons (D2-MSNs) can alleviate hyperalgesia, whereas activation of D2-MSNs aggravates pain, indicating that D2-MSNs are key cellular targets for neuropathic pain regulation (78, 91). D2R is also involved in the regulation of stress-induced analgesia; the analgesic effect induced by swimming stress can be blocked by D2 antagonist in the NAc, suggesting that NAc D2R mediates endogenous analgesic mechanism *via* the mesolimbic dopamine pathway (95). In the regulation of pain-emotion integration and comorbidity, NAc D2R-positive neurons regulate pain aversion by projecting to the ventral globus pallidus, and their dysfunction may mediate the comorbidity of neuralgia and depression, cooperating with signaling pathways such as CCR2 and A2AR (18, 26, 92). Clinical studies have further confirmed that reduced D2/D3 receptors availability in the ventral striatum (including NAc) of patients with chronic back pain is closely associated with the emotional dimension of pain and pain tolerance, further validating the regulatory role of NAc D2/D3 receptors in human chronic pain (96).

In summary, NAc D2R plays a key role in pain perception, emotional integration and pathological progression by regulating neuronal activity, signaling pathways, and neural circuits, which provides an important theoretical basis for targeted chronic pain therapy.

## 6 Anterior cingulate cortex

The anterior cingulate cortex (ACC) is a core region for the affective-unpleasantness dimension of pain, including suffering and the emotional response to nociceptive input (97). As depicted in Figure 1 (bottom panel), D1R and D2R in the ACC exert functional antagonism: D1R promotes pain signal transmission, whereas D2R inhibits it (98, 99). The balance between D1R and D2R signaling determines pain output.

### 6.1 The role of D1R in the ACC

Among the relevant neuromodulators, the D1R plays diametrically opposite roles in physiological and pathological conditions: it mediates analgesia under physiological conditions, whereas its function declines in chronic pain, thereby maintaining hyperalgesia. Consistent with Figure 1, D1R in the ACC is associated with pain facilitation *via* the cAMP/PKA pathway. In the physiological state, ACC D1R inhibits the excitability of pyramidal neurons by regulating HCN channels. Lançon et al. (19) found that dopamine through D1R signaling reduces the input resistance and discharge frequency of pyramidal neurons in layers II/III of the mouse ACC, and this cellular effect is associated with analgesia in animals (19). However, in chronic neuropathic pain, this D1R-mediated inhibition is significantly weakened and becomes a key factor in maintaining hyperalgesia. Notably, this functional change is not due to the altered D1R expression. Ortega-Legaspi et al. reported that D1R mRNA and protein levels in the ACC of rats with neuropathic pain showed no significant change, while D2R was upregulated, suggesting that the core pathology of D1R in chronic pain lies in reduced signal transduction efficiency (99). The function of D1R is also antagonistic to that of D2R. Liu et al. (98) optogenetically activated ACC D1R neurons and found that this aggravated trigeminal neuralgia, whereas activation of D2R neurons relieved pain, highlighting the fine division of labor of dopaminergic regulation (98). Beyond the sensory dimension, ACC D1R is also involved in the coding of pain-related emotions. Du et al. found that injection of the D1R agonist SKF-38393 into the ACC induced conditioned place avoidance (negative emotion), while the antagonist SCH-23390 alleviated anxiety-like behavior in the CFA inflammatory pain model (73). Navratilova et al. further confirmed that endogenous D1R signaling in the ACC is necessary for morphine-induced analgesia in the CeA (100). At the synaptic level, Darvish-Ghane et al. (16) knocked out ACC D1R, which led to the decreased mechanical pain threshold in mice, while D1R agonist SKF81297 or SKF38393 inhibited AMPAR-mediated excitatory postsynaptic currents (16). These findings suggest that activation of D1R primarily inhibits glutamatergic synaptic transmission in the ACC under the experimental conditions tested. However, its overall impact on ACC function may vary based on differences in cell types and neural circuits.

### 6.2 The role of D2R in the ACC

The expression and function of dopamine D2 receptor exhibits complex dynamics in different pain states. In the cg1 of ACC subregion of neuropathic pain rats, D2R mRNA and protein levels are significantly increased, and this upregulation is positively correlated with self-injurious behavior, suggesting that D2R is involved in the pathology of chronic pain (99). Regarding upstream inputs, dopaminergic projections from the VTA to the ACC inhibit mechanical nociceptive responses by activating postsynaptic D2R, constituting an endogenous descending analgesic pathway (98). More direct evidence comes from optogenetics: Liu et al. (98) selectively activated D2R neurons in the ACC, which significantly increased the mechanical pain threshold in the late stage of a chronic trigeminal neuralgia model and increased the time mice spent on the analgesic side in a real-time place preference test, indicating that D2R activation alleviates pain-related behavioral responses and promotes location preference, suggesting a dual impact on pain-related emotional states (98).

At the cellular level, D2R exerts analgesic effects by inhibiting excitatory transmission. *In vitro* electrophysiology has shown that dopamine inhibits AMPA currents in ACC pyramidal neurons *via* D2/D3 receptors, and reduces network excitability (101–103). However, this inhibition is significantly weakened under inflammatory pain conditions, suggesting that chronic pain can cause “dysfunction” of ACC dopaminergic signaling (104). Pharmacological studies have demonstrated that injecting dopamine or amantadine into the ACC alleviates chronic pain behavior (103). In the emotional dimension, ACC D2R also plays an important role. Sardari et al. found that a low dose of apomorphine alone did not affect the baseline pain threshold but significantly enhanced the combined analgesia effect of nicotine and morphine, and this synergistic effect was dependent on ACC dopamine receptors (105). Furthermore, studies on sleep deprivation have shown that activating D2R (but not D1R) in the ACC or NAc prevents sleep deprivation-induced hyperalgesia, further supporting the potential of D2R as an analgesic target (106).

## 7 The significance of the interaction between D1 and D2 receptors in pain

The modulation of pain by the dopaminergic system is not achieved through the isolated action of either D1 or D2 receptors, but rather relies on a delicate and dynamic interaction between the two. This interplay shapes the ultimate pain perception and behavioral output at the molecular, cellular, and circuit levels, encompassing patterns of functional synergy, functional antagonism, and state-dependent reorganization (107–109). Figure 1 highlights functional antagonism in the ACC (D1R promotes pain, D2R suppresses pain) and the opposing roles of D1-MSN and D2-MSN pathways in the NAc. Although the downstream signaling pathways of D1R (Gs-coupled) and D2R (Gi-coupled) are typically antagonistic, under specific conditions, they can be co-opted to produce potent analgesic effects (22, 110). Research by Holanda et al. provides direct evidence for this. In the mouse formalin test, the analgesia induced by either intrathecal or intracerebroventricular injection of neuropeptide S (NPS) could be completely blocked by either the D1R antagonist SCH23390 or the D2R antagonist raclopride alone (111). This indicates that the pain-relieving signal flow triggered by NPS requires simultaneous activation of both D1 and D2 receptor. This study reveals a novel downstream pain-relieving pathway that is dependent on the synergy of D1/D2 receptors, suggesting that in certain pharmacological contexts, the two are not “either-or” but rather “both indispensable” partners. In the NAc, D1R- and D2R-expressing MSNs have opposite effects on pain-related behaviors, suggesting the existence of a “push-pull mechanism” that regulates the intensity of pain signal perception within this circuit (78) (see also Figure 1, direct vs. indirect pathways). However, it is unclear whether this mechanism also applies to other pain-related brain regions. In the dorsal horn of the spinal cord, activation of D1R and D2R usually produces opposite effects. Studies have shown that intrathecal injection of D1R agonists promotes nociceptive responses, while D2R agonists produce analgesic effects (112, 113). This direct antagonism at the level of the sensory input gateway provides a fundamental mechanism for the rapid regulation of pain signal transmission intensity by the dopamine system. The ACC is a core brain region for the emotional component of pain (114). Lançon et al. (19) found that in the neuropathic pain state, the dopaminergic inhibitory control mediated by D2R in the ACC is weakened, leading to excessive neuronal excitation (19). Their pharmacological experiments showed that activating D2R or blocking D1R alleviates pain hypersensitivity. This suggests that in the ACC, the functions of D1R and D2R are mutually antagonistic: D1R promotes the processing of pain signals, while D2R inhibits it. Chronic pain disrupts this balance and potentially tilts it toward a D1R advantage. In the dorsal striatum, D1R and D2R are respectively expressed in the medium spiny neurons of the direct and indirect pathways, and the classical theory holds that their functions are mutually antagonistic (115). Magnusson et al. confirmed that activating D1R-MSNs promotes movement and may facilitate pain avoidance behavior, while activating D2R-MSNs inhibits movement and may hinder the expression of pain-related behaviors (116, 117). There is a two-way interaction between D1/D2 receptors and the opioid system; D1 receptors in the NAc can cooperate with the MOR receptors to enhance the analgesic effect (90, 118). Changes in the dopaminergic system induced by long-term pain may increase tolerance to opioids and increase the risk of addiction (see sections 5.1 and 5.2 for details). Table 1 summarizes functional differences of dopamine D1 and D2 receptors in different brain regions of pain (readers should note that the strength of evidence varies across regions and receptor subtypes, and some findings remain context-dependent).

**Table 1:** Summary of functional differences of dopamine D1 and D2 receptors in pain processing in different brain regions.

Brain region	Receptor type	Pain modulation	References
CeA	D1R	Complex and remains debated: may promote pain under certain conditions, or may inhibit pain and negative emotions	(14, 57)
	D2R	Suggested to exert analgesic effects; mediates VTA-CeA reward-analgesia pathway	(14, 15, 59)
PFC	D1R	Involved in pain signal integration and salience encoding; function declines in chronic pain	(66, 71, 72)
	D2R	Modulates nociceptive responses; context-dependent regulation	(11, 66, 80)
NAc	D1R	State-dependent: contributes to pain-reward balance; dysfunction in chronic pain	(78, 91, 92)
	D2R	Reported to exert analgesic effects; involved in inflammatory and neuropathic pain relief	(78, 91, 95)
ACC	D1R	Analgesic under physiological conditions; function declines in chronic pain	(19, 99, 100)
	D2R	May exert analgesic effects and contribute to endogenous pain relief mechanism.	(99, 100)

CeA the amygdala NAc the nucleus accumbens PFC the prefrontal cortex ACC the anterior cingulate cortex D1R/D2R D1 and D2 dopamine receptors.

## 8 Conclusion and prospect

Dopamine D1 and D2 receptors participate in the multidimensional regulation of pain through their extensive distribution and complex interactions in the central nervous system. Available evidence suggests that D1R is involved in opioid-analgesic effects within the NAc in certain contexts and contributes to pain-reward balance, whereas D2R has been implicated in the regulation of pain-related emotional processing in the ACC and CeA. Depending on the region of the brain, the pain state, and the behavioral context, D1 and D2 receptors may have opposite effects or may have synergistic effects. Together, these two effects contribute to the precise regulation of sensory and affective pain. Figure 1 summarizes these region-specific and pathway-specific mechanisms, providing a visual framework for understanding how D1R and D2R bidirectionally modulate pain. Several important limitations of the reviewed literature warrant acknowledgment. First, nearly all studies discussed are preclinical (rodent models); direct clinical evidence for D1R/D2R-specific mechanisms in chronic pain patients is extremely limited. Second, the reviewed studies predominantly employed pharmacological or optogenetic manipulations; causal evidence linking endogenous D1R/D2R signaling to specific pain dimensions remains sparse. Third, significant gaps remain in understanding (a) cell-type-specific expression patterns of D1R/D2R in pain-related circuits, (b) the functional relevance of D1/D2 receptor heteromers, and (c) sex-dependent differences in dopaminergic pain modulation. Fourth, the present review focused primarily on four brain regions (PFC, NAc, ACC, and amygdala) and did not systematically cover other relevant regions such as the periaqueductal gray (PAG), thalamus, or spinal dorsal horn. Future studies addressing these limitations will be essential for translating preclinical insights into clinical applications. Future research should explore several directions. First, the specific mechanisms of D1 and D2 receptors in different types of pain (neuropathic pain, inflammatory pain, cancer pain, etc.) require further clarification. Second, more selective receptor modulators that target specific brain regions or neuronal types need to be developed. Third, a better understanding of the interactions between the dopamine system and other systems (e.g., the immune and endocrine systems) in pain is necessary, particularly the recently discovered regulatory effects of the microbiota–gut–brain axis on the dopamine system and pain. The pain treatment strategy based on dopamine receptors may represent a promising approach for patients with chronic pain, especially those with severe emotional disorders and poor response to conventional therapies, though further clinical validation is needed.
